# Adenoid cystic carcinoma of the orbit without lacrimal gland involvement and with intracranial extension: A case report

**DOI:** 10.1016/j.radcr.2023.12.001

**Published:** 2023-12-28

**Authors:** Yu Ohkubo, Toshiyuki Okubo, Yusuke Asari, Yuki Matsuzaka, Mayuka Kitaguchi, Saori Shiota, Richi Fujita, Koichiro Okamoto, Hiroto Obata, Yukiko Kishida, Toshitaka Nagao

**Affiliations:** a Department of Radiology, University Hospital Mizonokuchi, Teikyo University School of Medicine, Kanagawa, Japan; b Department of Radiology, Tokyo Teishin Hospital, Tokyo, Japan; c Department of Radiology, Graduate School of Medicine, The University of Tokyo, Tokyo, Japan; d Department of Neurosurgery, Tokyo Teishin Hospital, Tokyo, Japan; e Department of Ophthalmology, Saitama Medical Center, Saitama Medical University, Kawagoe, Japan; f Department of Pathology, Tokyo Teishin Hospital, Tokyo, Japan; g Department of Anatomic Pathology, Tokyo Medical University, Tokyo, Japan

**Keywords:** Adenoid cystic carcinoma, Orbit, Lacrimal gland, Ectopic, Intracranial, Magnetic resonance imaging, Case report

## Abstract

We present a 76-year-old female with a 6-year history of decreased vision in the right eye and right-sided facial neuralgia. She had a T1 isointense and T2 isointense enhancing lesion in the right orbit and the middle cranial fossa on MRI examination. Granulomatous disease or meningioma was suspected, however, after removal, the tumor was identified by pathology as adenoid cystic carcinoma (ACC). The tumor has no radiological and clinical lacrimal grand involvement. ACC shows a slow and indolent growth pattern but is associated with poor long-term outcomes, mainly due to perineural invasion, local control failure, and distant metastasis. This case highlights the importance of a pathologic diagnosis and early intervention in similar presentations.

## Introduction

Adenoid cystic carcinoma (ACC) is an uncommon tumor, accounting for about 1% of all head and neck malignancies, and about 10% of all tumors of the salivary glands. It is the most commonly reported malignant tumor of the minor salivary glands and is also one of the most common cancers of the major salivary glands (the parotid, submandibular, and sublingual salivary glands). ACC can also involve lacrimal and ceruminous glands as well as other sites in the head and neck, including the nasal and paranasal sinuses, trachea, and larynx [1]. Orbital ACC is rare, most commonly originating from the lacrimal gland. Primary orbital ACC without lacrimal gland involvement has only been described in a small number of reports to our knowledge [2], [3], [4], [5], [6], [7], [8], [9]. We report a case of right orbital ACC without macroscopic lacrimal gland involvement showing skull base invasion and intracranial extension.

### Case presentation

**Fig. 1:**
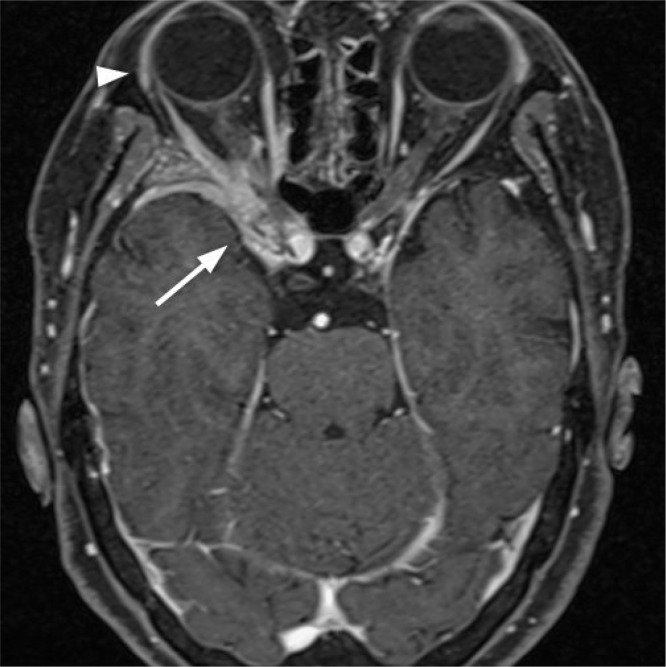
Fat-suppressed, postcontrast MR T1-weighted image shows an enhancing intraorbital mass (arrow) with no evidence of lacrimal involvement (arrowhead).

A 76-year-old female presented to our emergency department complaining of headache, nausea, and vomiting. She had a 6-year history of decreased right visual acuity and right-sided facial neuralgia. MRI examination revealed a mass in the right orbit with no evidence of lacrimal involvement (Fig. 1). Whole-body positron emission tomography (PET) scan showed no other systemic lesions. Biopsy and surgery were considered, but she declined them. Right visual acuity decreased gradually, was almost lost 3 years before her visit. She was followed up in our neurosurgical department.

On examination at emergency room, she had no light perception in the blind right eye with an afferent pupillary defect, and her visual acuity was measured at 20/40 in the left eye. There was 5 mm right proptosis. She had decreased sensation in the right trigeminal nerve area. Her laboratory investigations were unremarkable.

**Fig. 2:**
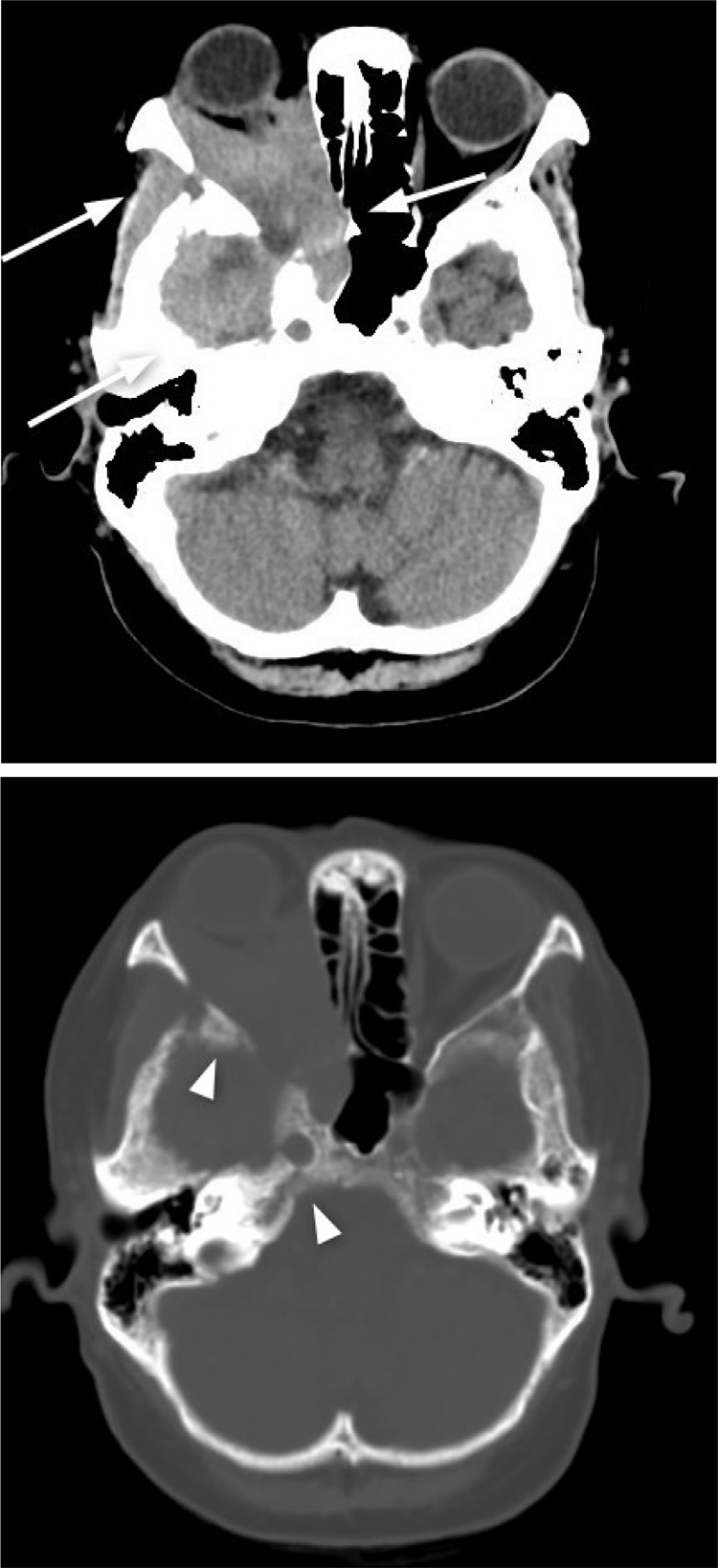
CT image demonstrates a mildly high-density lesion (arrows, A) occupying most of the right orbit extending to sphenoid sinus, skull base, and middle cranial fossa with bone erosion (arrowheads, B). There was no evident calcification.

CT imaging showed a mildly high-density lesion occupying most of the right orbit extending to neighboring regions with no evident calcification (Fig. 2).

**Fig. 3:**
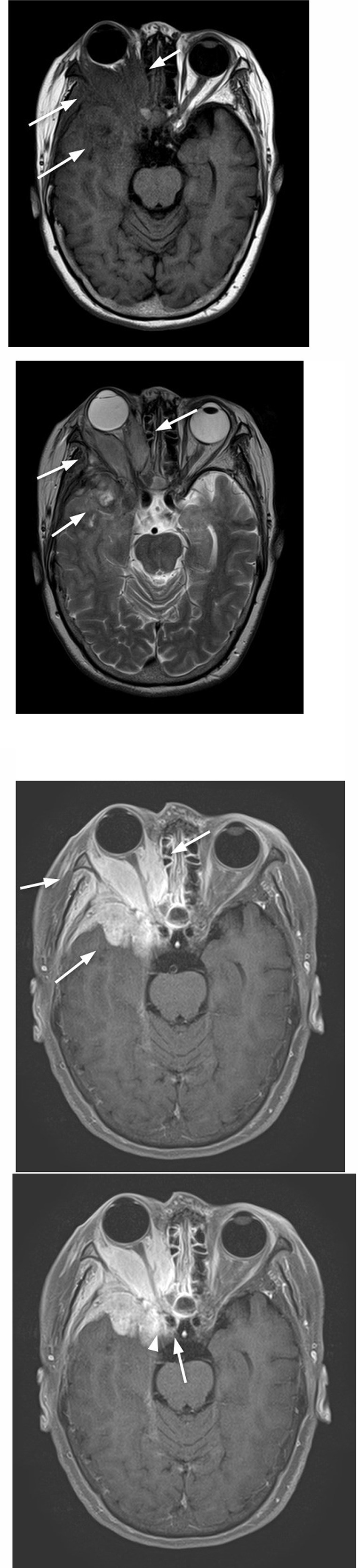
On the T1-weighted image, the mass is isointense compared to extraocular muscles and hypointense compared to orbital fat (arrows, A), hyperintense compared to extraocular muscles and hypointense compared to orbital fat on the T2-weighted image, (arrows, B). On contrast-enhanced fat-suppressed T1-weighted image, homogenous enhancement is shown (arrows, C). The mass encases the right internal carotid artery (arrows) and cavernous sinus (arrowheads) (D).

**Fig. 4:**
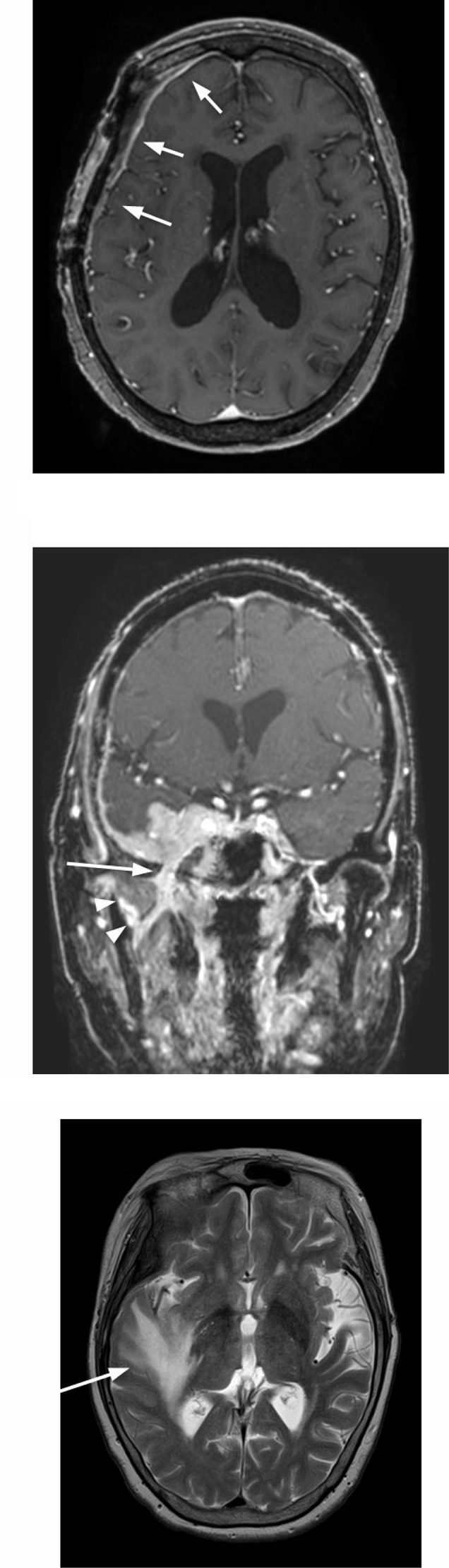
On contrast-enhanced, fat-suppressed, and T1-weighted images, dural thickening and enhancement (arrows)are also observed (A). Extracranial development via foramen ovale (arrows) and infiltration into pterygoid muscle (arrowheads) are demonstrated (B). On the T2-weighted image, cerebral edema in the right frontotemporal robe is remarkable (arrows, C).

On MRI imaging, the mass was isointense compared to extraocular muscles and hypointense compared to orbital fat on T1-weighted image (Fig. 3A), hyperintense compared to extraocular muscles and hypointense compared to orbital fat on T2-weighted image (Fig. 3B), Homogenous enhancement on Gadolinium contrast was shown (Fig. 3C). It encased the right internal carotid artery and cavernous sinus (Fig. 3D). Dural thickening and enhancement were also observed (Fig. 4A). Extracranial development via foramen ovale and infiltration into pterygoid muscle was suspected (Fig. 4B). Cerebral edema in the right frontotemporal robe was remarkable (Fig. 4C). Granulomatous disease or meningioma was suspected.

She underwent frontotemporal craniotomy and superior orbitotomy with subtotal resection of the tumor. Portions of the tumor encasing the internal carotid artery and cavernous sinus could not be removed.

**Fig. 5:**
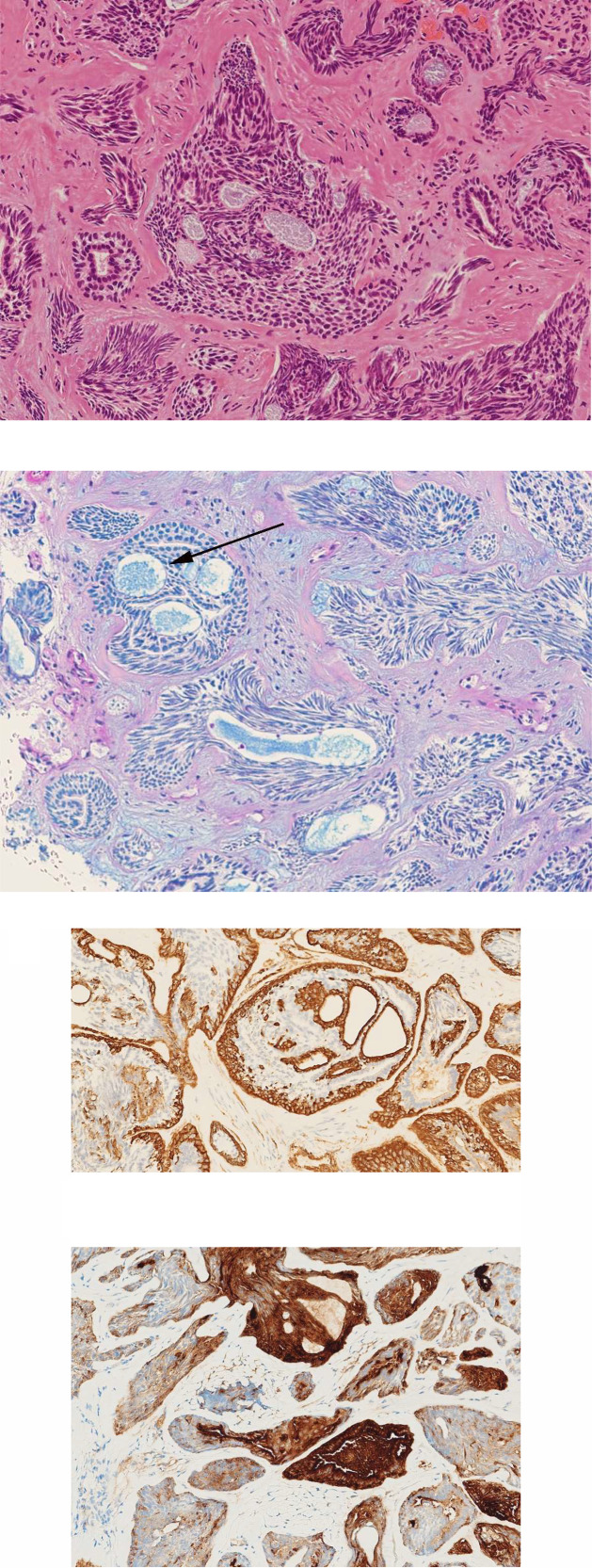
(A) (original magnification × 100, Hematoxylin and Eosin): The appearance of the tumor showing typical Swiss cheese configuration. Solid islands of epithelial and myoepithelial cells with a partly cribriform pattern are seen. (B) (original magnification × 100, Alcian-blue-PAS): The prominent staining highlights mucinous material in the glandular spaces (arrow). (C) (original magnification × 100, Smooth Muscle Actin), (D) (original magnification × 100, Epithelial Membrane Antigen): The tumor cells show predominantly biphasic ductal differentiation, partly cribriform pattern, and depict expression and reactivity to the corresponding confirmatory immunohistochemistry markers.

Histopathological examination showed solid islands of biphasic (epithelial and myoepithelial) ductal cells with a partly cribriform pattern, which was compatible with ACC (Fig. 5).

Postsurgical radiation therapy (Intensity Modulated Radiation Therapy = IMRT, total dose of 54 Gy in 30 fractions) was added.

At 6 months of follow-up, she denied headache or nausea and MRI showed stable residual tumor.

## Discussion

ACC is a rare malignant epithelial tumor that accounts for 1% of all malignant neoplasms of the head and neck and 10% of salivary gland malignancies. The age of onset is usually between 50 and 60 years, and no significant differences are found based on sex. ACC of the head and neck presents a slow and indolent growth pattern with an optimistic 5-year overall survival rate of 68%-90%. However, long-term outcomes revealed a decline in the 10- and 15-year overall survival rates of 52% and 28%, mainly due to perineural invasion, local control failure, and distant metastasis [10].

Orbital ACC commonly arises from the lacrimal gland, However, a small number of cases of primary orbital ACC without lacrimal gland involvement have been reported. Five cases involved the orbital apex with intracranial extension [2], [3], [4], [5], [6], 2 cases involved the medial orbit [7,8], and 1 case involved the inferior orbit [9].

Several hypotheses as to the origin of intraorbital ACC have been proposed, including the possibility of the tumor originating from ectopic lacrimal gland tissue, perineural spread of occult ACC from other regions of the head and neck, or hematogenous metastases from an unidentified distant location [6].

In our case, the origin of the tumor is unclear. Though a lacrimal gland biopsy was not performed, there was no evident lacrimal involvement on the first MRI examination (Fig. 1). Whole-body PET scan showed no other tumor site, suggesting that the origin of the tumor is located in the orbit.

The optimal treatment of orbital ACC is not established. Bin-Alamer et al. [11] reported that surgical resection was the most favored therapeutic strategy, and post-surgical radiotherapy could contribute to higher survival and lower recurrence rates. Systemic chemotherapy is mainly used as palliative treatment for metastatic and recurrent ACC and is only considered for preventing disease progression when further surgery and radiotherapy are impossible [10].

In summary, we report a rare case of orbital ACC with skull base invasion and intracranial extension. The tumor had no radiological or clinical lacrimal grand involvement. ACC shows a slow and indolent growth pattern but is associated with poor long-term outcomes, mainly due to perineural invasion, local control failure, and distant metastasis. This case highlights the importance of a pathologic diagnosis and early intervention in similar presentations.

## Patient consent

Informed consent to include the patient's information in the publication of this case report was obtained.

## References

[1] Coca-Pelaz A., Rodrigo J.P., Bradley P.J., Poorten V.V., Triantafyllou A., Hunt J.L. (2015). Adenoid cystic carcinoma of the head and neck – an update. Oral Oncol.

[2] Walsh R.D., Vagefi M.R., McClelland C.M., Alonso-Basanta M., Newman J.G., Farkas T. (2013). Primary adenoid cystic carcinoma of the orbital apex. Ophthalmic Plast Reconstr Surg.

[3] C. Macri, V. Juniat, G. Davis, D. Selva, Intraorbital and intracranial extension of adenoid cystic carcinoma without clinical or radiological lacrimal gland involvement, Orbit;41(6):797-801 10.1080/01676830.2021.1939731. 10.1080/01676830.2021.1939731 34107855

[4] Li B., Iordanous Y., Wang Y., Chakrabarti S., Allen L.H. (2016). Adenoid cystic carcinoma presenting as an orbital apex mass with intracranial extension. Can J Ophthalmol.

[5] Venkitaraman R., Madhavan J., Ramachandran K., Abraham E., Rajan B. (2008). Primary adenoid cystic carcinoma presenting as an orbital apex tumor. Neuro-Ophthalmol.

[6] Ghulaiga F.M.A., Alkhiary H., AlKhalidi H., Alkatan H.M. (2022). Adenoid cystic carcinoma of the orbit with bilateral cavernous sinus extension: a case report. Int J Surg Case Rep.

[7] Nava-Castaneda A., Kahuam-Lopez N., De La Fuente Díez Y., Velasco Y., Levy A., Sanchez-Bonilla F.G. (2021). Primary adenoid cystic carcinoma arising from an ectopic lacrimal gland involving both nasal orbits: a rare clinical entity. Orbit.

[8] Shields J.A., Shields C.L., Jr R.C.E., Ba J.A., Potter P.De (1997). Adenoid cystic carcinoma developing in the nasal orbit. Am J Ophthalmol.

[9] Lin S.C., Kau H.C., Yang C.F., Yang M.H., Tsai C.C., Kao S.C. (2008). Adenoid cystic carcinoma arising in the inferior orbit without evidence of lacrimal gland involvement. Ophthal Plast Reconstr Surg.

[10] Fang Y, Peng Z, Wang Y, Gao K, Liu Y, Fan R (2022). Current opinions on diagnosis and treatment of adenoid cystic carcinoma. Oral Oncol.

[11] Bin-Alamer O., Haider A.S., Chaudhary A., Balasubramanian K., Breeding T., Palmisciano P. (2022). Adenoid cystic carcinoma (ACC) infiltrating the skull base: a systematic review of clinical characteristics and management strategies. Cancer Diagn Progn.

